# White matter extension of the Melbourne Children's Regional Infant Brain atlas: M‐CRIB‐WM

**DOI:** 10.1002/hbm.24948

**Published:** 2020-02-21

**Authors:** Bonnie Alexander, Joseph Yuan‐Mou Yang, Sarah Hui Wen Yao, Michelle Hao Wu, Jian Chen, Claire E. Kelly, Gareth Ball, Lillian G. Matthews, Marc L. Seal, Peter J. Anderson, Lex W. Doyle, Jeanie L. Y. Cheong, Alicia J. Spittle, Deanne K. Thompson

**Affiliations:** ^1^ Developmental Imaging Murdoch Children's Research Institute Melbourne Victoria Australia; ^2^ Victorian Infant Brain Studies Murdoch Children's Research Institute Melbourne Victoria Australia; ^3^ Neuroscience Research Murdoch Children's Research Institute Melbourne Victoria Australia; ^4^ Department of Neurosurgery Royal Children's Hospital Melbourne Victoria Australia; ^5^ Department of Paediatrics The University of Melbourne Melbourne Victoria Australia; ^6^ Monash School of Medicine, Faculty of Medicine, Nursing and Health Sciences Monash University Clayton Victoria Australia; ^7^ Medical Imaging Royal Children's Hospital Melbourne Victoria Australia; ^8^ Department of Medicine Monash University Melbourne Victoria Australia; ^9^ Department of Pediatric Newborn Medicine, Brigham and Women's Hospital Harvard Medical School Boston Massachusetts; ^10^ Turner Institute for Brain and Mental Health, School of Psychological Sciences Monash University Melbourne Victoria Australia; ^11^ Newborn research Royal Women's Hospital Melbourne Victoria Australia; ^12^ Department of Obstetrics and Gynaecology The University of Melbourne Melbourne Victoria Australia; ^13^ Department of Physiotherapy The University of Melbourne Melbourne Victoria Australia; ^14^ Florey Institute of Neuroscience and Mental Health Melbourne Victoria Australia

**Keywords:** atlas, MRI, neonatal, parcellation, white matter

## Abstract

Brain atlases providing standardised identification of neonatal brain regions are key in investigating neurological disorders of early childhood. Our previously developed Melbourne Children's Regional Infant Brain (M‐CRIB) and M‐CRIB 2.0 neonatal brain atlases provide standardised parcellation of 100 brain regions including cortical, subcortical, and cerebellar regions. The aim of this study was to extend M‐CRIB atlas coverage to include 54 white matter (WM) regions. Participants were 10 healthy term‐born neonates that were used to create the initial M‐CRIB atlas. WM regions were manually segmented based on *T*_2_ images and co‐registered diffusion tensor imaging‐based, direction‐encoded colour maps. Our labelled regions imitate the Johns Hopkins University neonatal atlas, with minor anatomical modifications. All segmentations were reviewed and approved by a paediatric radiologist and a neurosurgery research fellow for anatomical accuracy. The resulting neonatal WM atlas comprises 54 WM regions: 24 paired regions, and six unpaired regions comprising five corpus callosum subdivisions, and one pontine crossing tract. Detailed protocols for manual WM parcellations are provided, and the M‐CRIB‐WM atlas is presented together with the existing M‐CRIB cortical, subcortical, and cerebellar parcellations in 10 individual neonatal MRI data sets. The novel M‐CRIB‐WM atlas, along with the M‐CRIB cortical and subcortical atlases, provide neonatal whole brain MRI coverage in the first multi‐subject manually parcellated neonatal atlas compatible with atlases commonly used at older time points. The M‐CRIB‐WM atlas is publicly available, providing a valuable tool that will help facilitate neuroimaging research into neonatal brain development in both healthy and diseased states.

**Abbreviations**
AAL: automated anatomical labeling
ACR: anterior corona radiata
ALIC: anterior limb of internal capsule
ANTs: advanced normalization tools
AP: anterior–posterior
BET: brain extraction tool
CC: corpus callosum
CGC: cingulum cingular part
CGH: cingulum hippocampal part
CP: cerebellar peduncle
CR: corona radiata
CST: corticospinal tracts
DEC: direction‐encoded color
DTI: diffusion tensor imaging
DWI: diffusion weighted images
EC: external capsule
EPI: echo planar imaging
FLIRT: Functional Magnetic Resonance Imaging of the Brain's Linear Image Registration Tool
FMRI: functional magnetic resonance imaging
FOV: field of view
FSL: functional magnetic resonance imaging Software Library
FUGUE: FMRIB's utility for geometrically unwarping echo planar images
Fx: fornix
GM: grey matter
IC: internal capsule
ICP: inferior cerebellar peduncle
IFO: inferior fronto‐occipital fasciculus
ITK: Insight Toolkit
JHU: Johns Hopkins University
LR: left–right
MCP: middle cerebellar peduncle
M‐CRIB: Melbourne Children's Regional Infant Brain
M‐CRIB‐WM: Melbourne Children's Regional Infant Brain‐white matter
ML: medial lemniscus
MRI: magnetic resonance imaging
PCR: posterior corona radiata
PCT: pontine crossing tract
PLIC: posterior limb of internal capsule
PTR: posterior thalamic radiation
RLIC: retrolenticular part of internal capsule
SCP: superior cerebellar peduncle
SCR: superior corona radiata
SFO: superior fronto‐occipital fasciculus
SI: superior–inferior
SLF: superior longitudinal fasciculus
SS: sagittal stratum
ST: stria terminalis
TAP: tapetum
TE: echo time
TR: repetition time
UFC: uncinate fasciculus
WM: white matter

## 1 INTRODUCTION

Parcellated brain atlases are a key component of many neuroimaging tools. They can facilitate identification and labelling of brain regions in a consistent manner, such that properties of these regions can be compared across brains and across time points. Until recently, few parcellated atlases were available for the crucial neonatal time period where the foundations for all future neurodevelopment are set. Various perinatal events and conditions, such as very preterm birth, congenital heart disease, neonatal encephalopathy, or stroke, may be associated with alterations to white matter (WM) development and in turn adverse neurodevelopmental outcomes (Anderson, Cheong, & Thompson, 2015; Dubois et al., 2014). Investigating properties of individual WM regions, such as volume, shape and surface area, microstructure, and myelination, and their behavioural and clinical correlates in typically and atypically developing populations, is therefore of potential clinical relevance (e.g., Mori 2009; Oishi et al., 2009). The provision of WM atlases at the neonatal time point is critical for investigating both typical and atypical WM development.

During the neonatal period, MRI images have relatively low spatial resolution due to small brain size and have different tissue contrast compared with older children and adults due to partial myelination and dynamic tissue properties in neonates (Heemskerk et al., 2013). Over the last decade, increasing efforts in the neonatal brain imaging field have led to development of several neonatal parcellated atlases (Alexander et al., 2017; Alexander et al., 2019; Blesa et al., 2016; de Macedo Rodrigues et al., 2015; Feng et al., 2019; Gousias et al., 2012; Kuklisova‐Murgasova et al., 2011; Makropoulos et al., 2016; Oishi et al., 2011; Shi et al., 2010; Shi et al., 2011). These atlases differ in image modality and quality, parcellation technique, and parcellation schemes. Many of these atlases were defined on *T*_2_‐weighted images (which provide higher tissue contrast than *T*_1_‐weighted images due to partial myelination at the neonatal time point) and focus on parcellation of cortical regions and deep grey nuclei. WM segmentation has generally been provided as a single label or a few regions (Alexander et al., 2017; Alexander et al., 2019; de Macedo Rodrigues et al., 2015), or included together with adjacent grey matter (GM) in parcellated regions (Gousias et al., 2012; Shi et al., 2011; Tzourio‐Mazoyer et al., 2002). The major WM tracts are extant at term (Dubois et al., 2014), however, these cannot be defined based on *T*_1_‐ or *T*_2_‐weighted images alone. In order to delineate anatomical tracts within WM, diffusion weighted images (DWI), which provide information about WM fibre orientation, are required.

One atlas to date, the ‘*JHU‐neonate‐SS*’ atlas (Oishi et al., 2011) has provided manually delineated anatomical WM regions using neonatal DWI data. The atlas consists of voxel‐wise averaging of 122 parcellated brain regions altogether, including 52 WM regions, from the MRI data of 25 healthy neonates. The WM regions were manually segmented based on a single participant's MRI data set, which was then warped to the group‐averaged brain template. The parcellated detail of the *JHU‐neonate‐SS* atlas is unprecedented and demonstrates the ability of the included regions to be delineated at term. This has allowed properties of these WM regions such as diffusion metrics and connectivity to be studied in neonates (e.g., Chang et al., 2016: Pannek, Hatzigeorgiou, Colditz, & Rose, 2013). Importantly, the *JHU‐neonate‐SS* atlas provides standardised identification of regions at the neonatal time point and compatibility with the adult JHU atlas (Mori et al., 2008), such that regional properties can be compared across developmental time points. However, a training set comprising a single participant does not contain individual variability in brain morphology.

Accounting for individual anatomical variance is an important factor to consider when studying a period of brain development that is marked by significant brain structural changes and growth (Shi et al., 2011). Some endeavours have been made to address this issue by warping existing single‐subject atlases to multiple neonatal subjects, with the aim of providing larger atlas training sets with greater inter‐subject variability. For example, Shi et al. (Shi et al., 2011) warped the adult parcellated ‘*Automated Anatomical Labelling*’ (AAL) atlas (Tzourio‐Mazoyer et al., 2002) (defined based on *T*_1_ images) to an infant longitudinal sample. However, it is generally acknowledged that warping adult atlases to infant space offers limited accuracy, due to morphological differences between the adult brain and the developing neonatal brain (Alexander et al., 2017; Blesa et al., 2016; Dickie et al., 2017; Fillmore, Richards, Phillips‐Meek, Cryer, & Stevens, 2015; Kazemi, Moghaddam, Grebe, Gondry‐Jouet, & Wallois, 2007; Richards, Sanchez, Phillips‐Meek, & Xie, 2016; Sanchez, Richards, & Almli, 2012). Additionally, warping a single parcellated image to multiple participants, even those of the same age, is likely to introduce labelling error related to imperfect registration aligning different target brains. This occurs in instances where there are individual differences in morphology or image properties between the template and the target brains (Akhondi‐Asl, Hoyte, Lockhart, & Warfield, 2014). Target brains that differ more greatly from the template will incur more marked registration error, and thus a multi‐subject training set that captures some of the individual variability in the population is valuable. The ‘gold standard’ procedure for defining an accurate and broadly applicable parcellated atlas is manual segmentation in a large sample of representative individuals (Gousias et al., 2012; Shi et al., 2010). In adults, training sets comprising 10 individuals have been found sufficient to optimise parcellation based on label fusion algorithms (Heckemann, Hajnal, Aljabar, Rueckert, & Hammers, 2006) and optimise structural templates to represent morphological variability in the population (Croxson, Forkel, Cerliani, & Thiebaut de Schotten, 2018); with additional subjects providing diminishing (Heckemann et al., 2006) or no (Croxson et al., 2018) benefit. Although equivalent studies do not yet appear available in neonates, this provides a baseline indication of sample size from which to begin in neonatal data, while acknowledging the potentially greater variability in morphology observed at this time point.

We previously presented the *Melbourne Children*'*s Regional Infant Brain* (M‐CRIB) atlas, a multi‐subject (*N* = 10), manually parcellated atlas of cortical, basal ganglia, thalamic, cerebellar, and other subcortical regions (Alexander et al., 2017) that is compatible with the *Desikan‐Killiany* adult cortical atlas (Desikan et al., 2006) and some subcortical regions automatically segmented in *FreeSurfer*. Compatibility of neonatal atlases with those commonly used at older timepoints is important for longitudinal investigations of brain development and for tracking the progression of developmental disorders (de Macedo Rodrigues et al., 2015; Gousias et al., 2012; Oishi et al., 2011). There is currently an unmet need for a multi‐subject, manually parcellated neonatal WM atlas to provide standardised identification of WM regions in a way that is compatible with atlases commonly used at older time points. The aim of this study was to extend the coverage of the M‐CRIB atlas to include manually parcellated WM regions by utilising neonatal DWI data. We elected to model our parcellation scheme on that provided by the *JHU‐neonate‐SS* atlas (Oishi et al., 2011), which has label nomenclature and terminology consistent with the commonly used adult JHU atlas (Mori et al., 2008; Mori, W., Nagae‐Poetscher, & van Zijl, 2005; Oishi et al., 2009). In this article, we detail a manual WM parcellation scheme in 10 term‐born neonates, presented as a WM extension of the M‐CRIB atlas, which we have named the M‐CRIB‐WM atlas. Together with the M‐CRIB cortical and subcortical atlases, we provide the first detailed multi‐subject neonatal atlas encompassing whole brain MRI coverage, including extensive standardised GM and WM parcellations.

## 2 MATERIALS AND METHODS

### 2.1 Participants

Participants were 10 healthy term‐born neonates (≥37 weeks' gestation; four females; gestational age at scanning 40.29–43.00 weeks, *M* = 41.71, *SD* = 1.31). These participants were the same as those utilised for our existing M‐CRIB atlases (Alexander et al., 2017; Alexander et al., 2019). The participants were initially selected from a larger cohort of control infants with MRI scans, recruited as part of preterm studies (Spittle et al., 2014; Walsh, Doyle, Anderson, Lee, & Cheong, 2014). The 10 images included in this study were chosen on the basis of minimal motion and other artefact on *T*_2_‐ weighted images (Alexander et al., 2017). Neonates who received resuscitation at birth, were admitted to a neonatal intensive care or special care unit, had a birth weight of less than 2.5 kg, or had congenital conditions affecting growth and development were excluded (Spittle et al., 2014; Walsh et al., 2014). All 10 participants selected were assessed at age 2 years and did not have any major health problems, cerebral palsy or major cognitive delay (Alexander et al., 2017).

This study was approved by the Royal Children's Hospital Human Research Ethics Committees. Informed parental/guardian consent was obtained prior to the study commencement.

### 2.2 MRI data acquisition and preprocessing

Neonatal MRI scans were acquired at the Royal Children's Hospital, Melbourne, Australia, on a 3‐Tesla Siemens MAGNETOM Trio Tim scanner. Participants were scanned during nonsedated natural sleep. They were first fed, swaddled and fitted with ear plugs and ear muffs throughout the MRI study. Transverse *T*_2_ restore turbo spin echo sequences were acquired with 1 mm axial slices, flip angle = 120°, repetition time (TR) = 8,910 ms, echo time (TE) = 152 ms, field of view (FOV) = 192 × 192 mm^2^, matrix = 384 × 384, and in‐plane resolution 1 mm^2^ (automatically zero‐filled interpolated in image reconstruction to 0.5 × 0.5 × 1 mm). DWI sequences were acquired using a multi‐*b*‐value, single‐shot echo planar imaging (EPI) sequence with TR = 20,400 ms, TE = 120 ms, FOV = 173 × 173 mm^2^, matrix = 144 × 144, 100 axial slices, 1.2 mm isotropic voxels, 45 noncollinear gradient directions, *b*‐values ranging from 100 to 1,200 s/mm^2^, and 3 *b* = 0 s/mm^2^ volumes. The total diffusion sequence was divided into three separate acquisitions to improve compliance, and if any of the diffusion acquisitions had unacceptable levels of motion artefact, the scan was repeated whenever possible until acceptable diffusion images were acquired. All infants were scanned with the same diffusion sequence, including the same range of *b*‐values.

The *T*_2_ images were preprocessed prior to manual segmentation of the original M‐CRIB atlas (Alexander et al., 2017; Loh et al., 2016) and did not undergo any further preprocessing here. For the existing preprocessing, images were bias corrected using N4ITK (http://www.itk.org, RRID:SCR_001149) (Tustison et al., 2010), and skull‐stripped using the Functional MRI of the Brain (FMRIB) Software Library (FSL; http://www.fmrib.ox.ac.uk/fsl/, RRID:SCR_002823) Brain Extraction Tool (BET) (Smith, 2002). They were then aligned to anterior commissure‐posterior commissure line using 3D Slicer to correct for any head tilt, which could adversely affect delineation, thus allowing equivalent viewing perspectives of structures bilaterally; and resampled to 0.63 mm isotropic (calculated as the cube root of the 0.5 × 0.5 × 1 voxel size in order to obtain isotropic voxels while preserving voxel volume) using the FMRIB's Linear Image Registration Tool (FLIRT) (Greve & Fischl, 2009; Jenkinson, Bannister, Brady, & Smith, 2002; Jenkinson & Smith, 2001).

The DWI data were corrected for head motion and eddy current‐induced distortions using the FSL “eddy_correct” tool (Jenkinson & Smith, 2001), incorporating *b*‐vector reorientation (Leemans & Jones, 2009). Echo planar image distortions were corrected based on a gradient echo field map and FMRIB's Utility for Geometrically Unwarping Echo planar images (FUGUE), as previously described (Thompson et al., 2018). The diffusion tensor imaging (DTI) model was fitted using the weighted linear least squares method in FSL. The *b* = 0 s/mm^2^ images were first linearly registered to the *T*_2_ image using FLIRT (Greve & Fischl, 2009; Jenkinson et al., 2002; Jenkinson & Smith, 2001), followed by nonlinear registration to the same *T*_2_ image using Advanced Normalisation Tools (ANTs; http://www.picsl.upenn.edu/ANTS/, RRID:SCR_004757) (Avants et al., 2011; Avants, Epstein, Grossman, & Gee, 2008), with symmetric diffeomorphic normalisation as the transformation type and cross correlation as the similarity metric. The concatenated linear and nonlinear transformation matrices derived from both registration steps were applied to the tensor image (including reorientation of the tensor vectors), using the ANTs “*Reorient tensor image*” option, with linear interpolation used in resampling. Then, the principal DTI eigenvector (representing the modelled single‐fibre orientation per voxel) was multiplied by the fractional anisotropy (FA) image to generate a direction‐encoded colour (DEC) map with the default colour scheme: the anterior–posterior (AP)‐oriented fibres encoded in green; left–right (LR)‐oriented fibres encoded in red; and superior–inferior (SI)‐oriented fibres encoded in blue.

### 2.3 Protocols for delineating the WM regional boundaries

We derived our parcellation scheme from the JHU neonatal atlas parcellation scheme (Oishi et al., 2011) with modifications to improve anatomical clarity of the following WM structures. First, we incorporated five corpus callosum (CC) vertical subdivisions based on Hofer' Classification (Hofer & Frahm, 2006) instead of a LR division, considering the interhemispheric nature of this commissural WM tract. We adopted Hofer's CC subdivision method instead of a three parts (genu, body, and splenium) vertical subdivision for two main reasons. First, the subdivision scheme proposed in Hofer's Classification is based on dividing the maximum AP length of the mid‐sagittal CC into specific proportions. This helps standardise our parcellation protocol, making the parcellation more likely to be reproducible between different MRI data sets. Second, Hofer's Classification has previously been applied to processing of neonatal data, including reconstructing CC tractography based on these five subdivisions (Thompson et al., 2011; Thompson et al., 2012). Next, we utilised a LR division for the middle cerebellar peduncle (MCP) and labelled the pontine crossing tract (PCT) as a singular region instead of a LR division. The MCP is anatomically a ‘paired’ (i.e., left and right) structure connecting the cerebellum to pons. It consists of cerebellar afferent fibres from contralateral pontine nuclei, with fibres lateralised to each cerebellar hemisphere and the cerebellar vermis. Centrally, the PCT label constitutes these crossing pontine fibres, thus a singular label is anatomically more appropriate than a LR division. Lastly, we excluded brainstem divisions in our parcellation scheme, because such regions would be sections containing remaining voxels outside of the existing subregions traced in the brainstem. Thus, they could not be combined to produce complete brainstem subsections, or anatomically meaningful regions. Instead, we used the existing brainstem label already included in the original M‐CRIB and M‐CRIB 2.0 atlases.

#### 2.3.1 Regions in the brainstem

##### Corticospinal tracts

*Description*: This parcellation defines the portion of the corticospinal tracts (CST) in the ventral pons (Ture, Yasargil, Friedman, & Al‐Mefty, 2000). They consist of SI‐oriented fibres (blue‐coloured on DEC map). *Relevant boundaries*: *Superior*: The midbrain‐pontine junction *Inferior*: The pontine‐medullary junction *Posterior*: The pontine crossing tract (PCT; described below).

##### Pontine crossing tract

*Description*: This parcellation consists of LR‐oriented fibres from the pontine nuclei in the ventral pons (red colour on DEC map). *Relevant boundaries*: *Superior*: The midbrain‐pontine junction. *Inferior*: The pontine‐medullary junction. *Anterior*: The CST (described above). *Posterior*: The Medial Lemniscus (ML; described below) (Mori et al., 2008).

##### Medial lemniscus

*Description*: This parcellation defines the portion of ML in the ventral pons. They consist of SI‐oriented fibres (blue colour on DEC map) (Mori et al., 2008). *Relevant boundaries*: *Superior*: The midbrain‐pontine junction. *Inferior*: The pontine‐medullary junction. *Anterior*: The PCT (described previously).

##### Superior cerebellar peduncle

*Description*: This structure contains efferent cerebellar fibres, connecting the cerebellum to the midbrain. It is most easily distinguished from between the level of the cerebellar nuclei and the midbrain using the DEC map (Mori et al., 2008). *Relevant boundaries*: *Superior*: It is marked by the superior cerebellar peduncle (SCP) decussation fibres (red coloured on the colour DEC map) at the level of the midbrain. (Mori et al., 2008). *Inferior*: The dentate nuclei of the cerebellum, located medio‐posteriorly to the middle cerebellar peduncle (MCP; described below).

##### Middle cerebellar peduncle

*Description*: This structure contains entirely afferent cerebellar fibres, connecting the cerebellum to the ventral pons. *Relevant boundaries*: *Posterior*: The dentate nuclei in the cerebellum. *Medial*: The CST, PCT, and ML parcellations in the ventral pons, best visualised on the axial plane.

##### Inferior cerebellar peduncle

*Description*: This structure contains the spinocerebellar fibre tracts, connecting the cerebellum to the medulla and spinal cord. *Relevant boundaries*: *Superior*: The level of the mid‐pons. *Inferior*: The dorsolateral aspect of the medulla (Hirsch et al., 1989).

#### 2.3.2 Projection Fibres

The projection fibres enter or exit the brain via the spinal cord by traversing through the following structures – from inferiorly to superiorly (for ascending afferent fibres; or in reverse order for descending efferent fibres): cerebral peduncle (CP), internal capsule (IC), and corona radiata (CR). The CP‐IC boundary was arbitrarily defined at the level of the anterior commissure (Mori et al., 2008). The IC‐CR boundary was arbitrarily defined at the axial level where the IC and external capsule (EC) merged (Mori et al., 2008). The IC can be identified on axial planes as the ‘bend‐shaped’ WM region located between the caudate nucleus, the lentiform nucleus, and the thalamus. It was arbitrarily divided into four parts: the anterior limb (ALIC), the genu, the posterior limb (PLIC), and the retrolenticular part (RLIC). The CR was arbitrarily divided into three parts: anterior (ACR), superior (SCR), and posterior corona radiata (PCR) (Mori et al., 2008).

##### Cerebral peduncle

*Description*: The IC converges into the CP, forming the ventral portion of the midbrain. *Relevant boundaries*: *Superior*: The CP‐IC plane at the anterior commissure level (Mori et al., 2008). *Inferior*: The midbrain‐pontine junction.

##### Anterior limb of internal capsule

*Description*: The ALIC is the anterior bend of the IC in front of the genu. We included the genu of IC into this parcellation – as per the approach adopted by the *JHU‐neonatal‐SS* atlas. *Relevant boundaries*: *Superior*: The IC‐CR plane against the ACR (Mori et al., 2008). *Inferior*: The CP‐IC plane (Mori et al., 2008). *Medial*: The head of the caudate nucleus. *Lateral*: The lentiform nucleus. *Posterior*: The PLIC (described below).

##### Posterior limb of internal capsule

*Description*: This structure represents the posterior bend of the IC, behind the genu. *Relevant boundaries*: *Superior*: The CR‐IC plane against the SCR. *Inferior*: The CP‐IC plane. *Anterior*: The genu of the IC. *Posterior*: The RLIC. This posterior boundary can be identified by a change in the dominant fibre orientation identified on the DEC map – from predominantly blue‐coloured SI‐oriented fibres in the PLIC to predominantly green‐coloured AP‐oriented fibres in the retrolenticular part of internal capsule (RLIC; described below). *Medial*: The thalamus. *Lateral*: The lentiform nucleus.

##### Retrolenticular part of internal capsule

*Description*: This portion of the IC is caudal to the lenticular nucleus and carries the optic radiation. *Relevant boundaries*: *Superior*: The CR‐IC plane against the PCR. *Inferior*: The sagittal stratum (SS) at the CP‐IC plane. *Anterior*: The PLIC. *Posterior*: The posterior thalamic radiation (PTR). This posterior boundary was arbitrarily defined by an imaginary vertical line extending from the midpoint of the CC splenium in the mid‐sagittal plane (Mori et al., 2008).

##### Anterior corona radiata

*Description*: The CR refers to the subcortical WM region inferior to the centrum semiovale and superior to the IC. *Relevant boundaries*: *Inferior*: The CR‐IC plane against the ALIC (Mori et al., 2008). *Posterior*: The superior corona radiata. This posterior boundary was defined arbitrarily by an imaginary vertical line extending from the posterior edge of the genu of CC in the mid‐sagittal plane (Mori & van Zijl, 2007).

##### Superior corona radiata

*Description*: Arbitrary boundaries were used to parcellate the SCR. *Relevant boundaries*: *Inferior*: The CR‐IC plane against the PLIC (described previously). *Anterior*: The ACR (described previously). *Posterior*: The PCR. This posterior boundary was defined arbitrarily by an imaginary vertical line extending from the anterior edge of the CC splenium in the mid‐sagittal plane (Mori et al., 2008; Mori & van Zijl, 2007).

##### Posterior corona radiata

*Description*: Arbitrary boundaries were used to parcellate the PCR. *Relevant boundaries*: *Inferior*: By the RLIC (anteriorly) and the PTR (posteriorly). This was defined arbitrarily by an imaginary horizontal line extending from the midpoint of the CC splenium in the mid‐sagittal plane (Mori et al., 2008). *Anterior*: The SCR (described previously). *Medial*: The forceps major and tapetum (TAP; described below) of the CC. *Lateral*: The superior longitudinal fasciculus (SLF; described below).

#### 2.3.3 Association Fibres

##### Cingulum cingular part

*Description*: This parcellation defines the frontal component of the cingulum WM within the cingulate gyrus. The cingulate gyrus is located immediately above the CC and below the cingular sulcus. It curves around the back of the CC splenium and continues as the hippocampal part of the cingulum (CGH) that enters the mesial temporal lobe (Shah, Jhawar, & Goel, 2012). *Relevant boundaries*: *Superior*: The cingular sulcus. *Inferior*: The CC. *Posterior*: This was arbitrarily delineated against the CGH by an imaginary horizontal line extending from the midpoint of the CC splenium in the mid‐sagittal plane (Mori et al., 2008). This division corresponds to a change in the dominant fibre orientation identified on the DEC map – from predominantly green‐coloured AP‐oriented fibres in the CGC to predominantly blue‐coloured SI‐oriented fibres in the CGH. *Lateral*: The CC body fibres.

##### Cingulum Hippocampal Part:

*Description*: The CGH courses within the parahippocampal gyrus and terminates anteriorly in the mesial temporal lobe (Shah et al., 2012). *Relevant boundaries*: *Superior*: The CGC (described previously). *Temporal terminations*: We arbitrarily defined this to be at the level of the hippocampal head in the sagittal plane.

##### Fornix

*Description*: This parcellation defines the forniceal crus, body, and column to the level of the anterior commissure (Nieuwenhuys, Voogd, & Van Hujizen, 2008; Shah et al., 2012). The precommissural column fibres to the septal region were not included due to limited image resolution. The forniceal body can be identified immediately inferiorly to the CC in the mid‐sagittal plane. *Relevant boundaries*: *Superior*: The body of CC. *Anterior and inferior*: At the level of the anterior commissure. *Posterior*: forniceal crus posterior to the thalamus.

##### Stria terminalis

*Description*: This WM tract is the main efferent fibre pathway from the amygdala that courses along the ventricular surface of the thalamus (Nieuwenhuys et al., 2008; Shah et al., 2012). This parcellation also includes the remaining portion of the forniceal crus and fimbria – differentiation of these two fibre tracts is not possible due to the current limited image resolution. The forniceal crus courses immediately posterior to the thalamus and medial to the SS (Mori et al., 2008). It continues caudally as the fimbria when entering the mesial temporal lobe (Nieuwenhuys et al., 2008; Shah et al., 2012). *Relevant boundaries*: *Anterior and superior*: the forniceal crus (included in the fornix label). *Temporal terminations*: We defined the fimbria terminations arbitrarily at two axial slices above the level of the CP, at the diencephalon–midbrain junction.

##### Superior longitudinal fasciculus

*Description*: This WM tract provides connections to the frontal, parietal, and temporal lobes (Martino et al., 2013). It is located dorsolaterally to the CR (Mori et al., 2008). *Relevant boundaries*: *Medial*: The fronto‐parietal component of the SLF (contains predominantly green‐coloured AP‐oriented fibres) is bounded medially by the CR (contains predominantly blue‐coloured SI‐oriented fibres). The temporal component of the SLF (contains predominantly blue‐coloured SI‐oriented fibres) is bounded medially by the PTR anteriorly and the SS posteriorly (both of which contain predominantly green‐coloured AP‐oriented fibres).

##### External capsule

*Description*: This parcellation includes both the EC and extreme capsule, with the intervening GM, the claustrum. Separating these regions is not possible due to limitations in the image resolution. It excludes the portion of the EC containing the inferior fronto‐occipital fasciculus (IFO) and the uncinate fasciculus (UFC), both of which are parcellated separately (described below). *Relevant boundaries*: *Superior*: The axial slice where the EC and IC merge. *Inferior*: An arbitrary boundary against the IFO – identified by the changes in the dominant fibre orientation from the DEC map (from predominantly SI‐oriented, blue‐coloured fibres in the EC to predominantly AP‐oriented, green‐coloured fibres in the IFO). *Medial*: The lentiform nucleus and the IC (Mori et al., 2008). *Lateral*: The insular cortex.

##### Posterior thalamic radiation

*Description*: This parcellation contains WM tracts that connect the caudal thalamus to the occipital and parietal lobes. The fibres are predominantly AP‐oriented, green‐coloured fibres on the DEC map, and are best visualised on the axial plane. *Relevant boundaries*: *Superior*: The PCR (described previously). *Inferior*: The SS (described below) (Mori et al., 2008). *Anterior*: The RLIC (described previously).

##### Sagittal stratum

*Description*: This WM region contains long association WM tracts, such as the IFO, optic radiation and PTR, with fibre projections to the occipital lobe. Division against the PTR parcellation is arbitrary. The SS was best visualised on the sagittal plane (Mori et al., 2008). *Relevant boundaries*: *Superior*: The RLIC (anteriorly) and the PTR (posteriorly). This superior boundary was arbitrarily defined at the anterior commissure level. *Anterior*: The IFO (described below) and the UFC (described below).

##### Superior fronto‐occipital fasciculus

*Description*: This WM tract, typically vestigial in humans, connects the occipital and frontal lobes and extends posteriorly along the dorsal edge of the caudate nucleus (Forkel et al., 2014; Jellison et al., 2004). *Relevant boundaries*: *Superior and lateral*: The SCR (described previously) (Mori et al., 2008). *Medial*: The head of caudate nucleus and the lateral ventricle.

##### Inferior fronto‐occipital fasciculus

*Description*: This WM tract forms the long‐ranged frontal and occipital connection (Jellison et al., 2004; Martino, Brogna, Robles, Vergani, & Duffau, 2010; Mori et al., 2008). This parcellation defines the portion of the IFO that traverses through the EC. *Relevant boundaries*: *Superior*: The EC. *Inferior*: The insular segment of the UFC at the temporal stem (Choi, Han, Yee, & Lee, 2010). Its boundary against the UFC was identified by the changes in the dominant fibre orientation from the DEC map (from predominantly AP‐oriented, green‐coloured fibres for the IFO to blue‐coloured SI‐oriented fibres for the UFC) (Ebeling & von Cramon, 1992; Kier, Staib, Davis, & Bronen, 2004) *Posterior*: The SS (described previously) (Jellison et al., 2004). *Medial*: The lentiform nucleus. *Lateral*: The insular cortex.

##### Uncinate fasciculus

*Description*: This hook‐shaped WM tract connects the orbitofrontal lobe to the anterior temporal lobe via the temporal stem portion of the EC (Ebeling & von Cramon, 1992; Jellison et al., 2004; Kier et al., 2004). This parcellation defines the insular segment of the UFC that traverses through the temporal stem (Choi et al., 2010). *Relevant boundaries*: *Superior*: The IFO (described previously). *Inferior*: The midbrain‐pontine junction. *Posterior*: The SS (described previously).

#### 2.3.4 Commissural Fibres

The CC provides the main inter‐hemispheric connections for the cerebrum. We did not parcellate the anterior and posterior commissures due to their small sizes in neonates and the limited image resolution. We adopted Hofer's classification, segmenting the CC into five divisions, based on its AP length at the mid‐sagittal plane (Hofer & Frahm, 2006). The TAP contains the CC temporal fibres and was parcellated as a separate region. The CC is best located in the mid‐sagittal plane, above the lateral ventricles and below the cingulate gyrus. The CR marks the lateral boundaries for the CC parcellations (Mori et al., 2008).

##### Corpus callosum I

*Description*: The CCI is the anterior 1/6 of the CC at the mid‐sagittal plane. It represents both the rostrum and the genu of the CC (Hofer & Frahm, 2006).

##### Corpus callosum II

*Description*: The CCII is defined as the portion of the CC between the anterior 1/2 and the anterior 1/6 of the CC at the mid‐sagittal plane. It represents the anterior body of the CC (Hofer & Frahm, 2006).

##### Corpus Callosum III

*Description*: The CCIII is between the posterior 1/2 and the posterior 1/3 of the CC at the mid‐sagittal plane. It represents the posterior body of the CC (Hofer & Frahm, 2006).

##### Corpus Callosum IV

*Description*: The CCIV is between the posterior 1/3 and the posterior 1/5 of the CC at the mid‐sagittal plane. It represents the isthmus of the CC (Hofer & Frahm, 2006).

##### Corpus Callosum V

*Description*: The CCV is the posterior 1/5 of the CC at the mid‐sagittal plane. It represents the splenium of the CC (Hofer & Frahm, 2006).

##### Tapetum

*Description*: The TAP is best located within the lateral ventricular wall at the level of ventricular trigone in the occipital lobe. It is medial to the SS and can be readily distinguished from the SS on the DEC map due to differences in the predominant fibre orientation (TAP contains predominantly blue‐coloured, SI‐oriented temporal CC fibres; the SS contains predominantly green‐coloured, AP‐oriented association fibres) (Mori et al., 2008).

### 2.4 Manual WM parcellation methods

All WM structures were delineated manually on the *T*_2_‐weighted images and co‐registered DEC maps in volume space using Insight Toolkit (ITK)‐SNAP v 3.6.0 (http://www.nitrc.org/projects/itk-snap/, RRID:SCR_002010) (Yushkevich et al., 2006), which simultaneously displays axial, sagittal, and coronal views along with a composite 3D surface representation of utilised labels.

To aid parcellation of regions containing boundaries between multiple adjacent WM tracts, we constructed individual Nearest Axis maps (see script in Appendix) where each voxel was assigned a value representing the nearest image axis (AP, LR, or IS) to its principal direction of diffusion, based on angular distance. These maps were used to clarify the region boundaries in situations where the primary fibre direction was not obvious via visual inspection of the DEC map. This creates artificial boundaries to help distinguish between voxels containing tracts running, for example, predominantly SI adjacent to voxels containing tracts running AP.

We overlaid the following parameter maps on *T*_2_‐weighted structural images and utilised them in a stepwise manner, in order to identify boundaries and remove boundary ambiguity between different WM regions. First, we overlaid the co‐registered DEC map, which revealed the principal fibre direction in each WM region. Many regions comprised some boundaries defined based on anatomical landmarks from the *T*_2_‐weighted images, and some boundaries based on diffusion direction from the DEC maps. Thus, in the first instance, delineating all boundaries of a region involved toggling between the *T*_2_‐weighted image and the DEC map. Next, the co‐registered Nearest Axis map was overlaid to aid boundary definition in cases where the DEC‐based anatomical boundary was ambiguous. Voxels were only selected if they had the nearest image axis to the principal fibre direction for the WM region, as shown on the Nearest Axis map. Then, the parcellation boundaries were further checked against the underlying *T*_2_‐weighted images to ensure that they conformed with any structural landmarks specified as boundaries.

Manual parcellation was performed and checked on a combination of axial, sagittal and coronal slices, leveraging the clearest perspective available for each WM structure. Parcellation was performed brain‐by‐brain, rather than region‐by‐region. This was considered most efficient and effective because boundaries of neighbouring structures are often interdependent, and the visibility of adjacent structures provided much insight in delineating the boundaries of the current parcellated region. In a few regions where anatomy was less clear due to small or subtle structures or partial voluming (e.g., as seen in the fornix), tracing in a single region was then checked in all brains and edited for consistency if needed. The CC was parcellated first in each brain, followed by the cingulum. The IC and EC were then traced. The IC segmentations provided shared boundaries for the PTR, SLF, and TAP.

Although all planes were used to view and check parcellations, some structures were primarily traced in a combination of the sagittal and coronal planes as these offered the clearest contrast/information, including the CC, cingulum, and fornix. Other structures were primarily traced in the axial plane, including the IC and EC, PTR, SLF, TAP, CP, SCP, MCP, ICP, CST, PCT, and ML. The coronal plane was used to cross check the accuracy of regional boundaries. The CP, SCP, MCP, and ICP are also intuitive to trace based on the *T*_2_ images.

Manual parcellation of all 54 WM structures was completed in all 10 individuals by one operator (S.Y.). A neurosurgery research fellow (J.Y., who has over 8 years of diffusion MRI research experience and over 6 years of neurosurgical practice), and a paediatric radiologist (M.W., who has over 10 years of paediatric radiology practice) confirmed the accuracy of each region's boundaries on all brains, based on the proposed parcellation scheme used in this study. Training for performing segmentations involved studying WM anatomy as pertaining to the JHU neonatal atlas and other literature cited in Section 2.3. When tracing was begun, for the first two brains, tracing was performed and the accuracy of each region was checked and adjusted if needed in consultation with J.Y.

**Figure 1:**
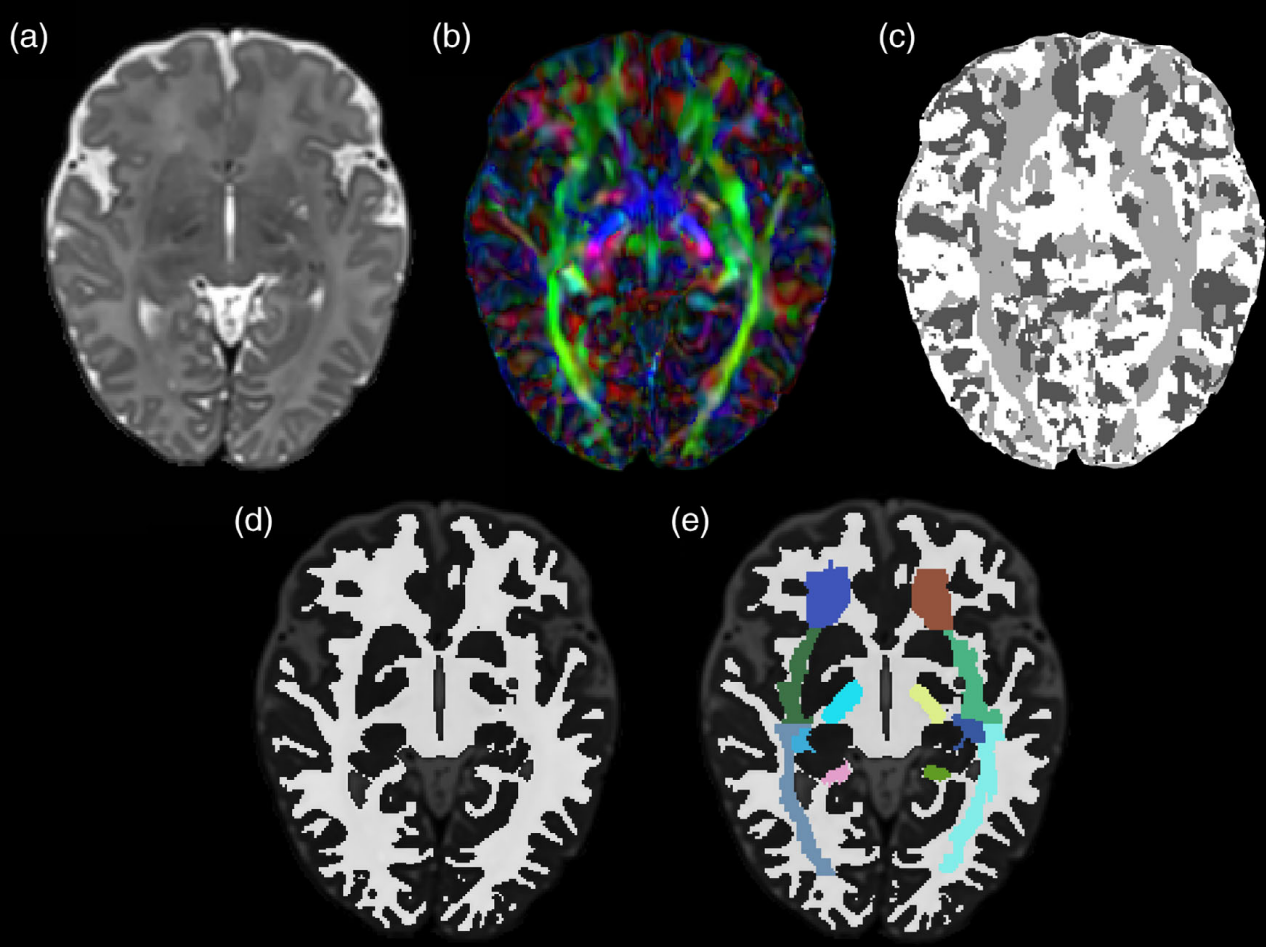
Illustration of the stepwise white matter (WM) parcellation process. (a) *T*_2_‐weighted image; (b) Direction encoded colour (DEC) map; (c) Nearest Axis map; (d) WM mask; (e) WM mask with parcellated regions overlaid. Images are displayed in radiological orientation

Parcellations for each individual were then masked using the same individual's WM tissue mask. This mask was created by combining and binarising the individual' WM‐containing labels from the M‐CRIB 2.0 (Alexander et al., 2019) *T*_2_‐based parcellated atlas; specifically, cerebral WM, CC, ventral diencephalon, brainstem, and cerebellar WM labels (Figure 1). The binarised WM mask was then multiplied by the parcellated WM atlas image using ‘fslmaths’ tools available as part of the FSL suite (Jenkinson, Beckmann, Behrens, Woolrich, & Smith, 2012; Smith et al., 2004). This allowed M‐CRIB‐WM parcellations to be combined with the whole‐brain M‐CRIB 2.0 atlas parcellations.

Manual parcellation of the 10 brains occurred part‐time over a period of 12 months. Training for WM regional boundary definition took approximately 160 hours. Manual label tracing took approximately 25 hours per brain. Reviewing all brain parcellations with an expert took approximately 20 hours, and subsequent editing to refine the parcellations from the 10 brains occupied approximately 30 hours in total.

## 3 RESULTS

**Figure 2:**
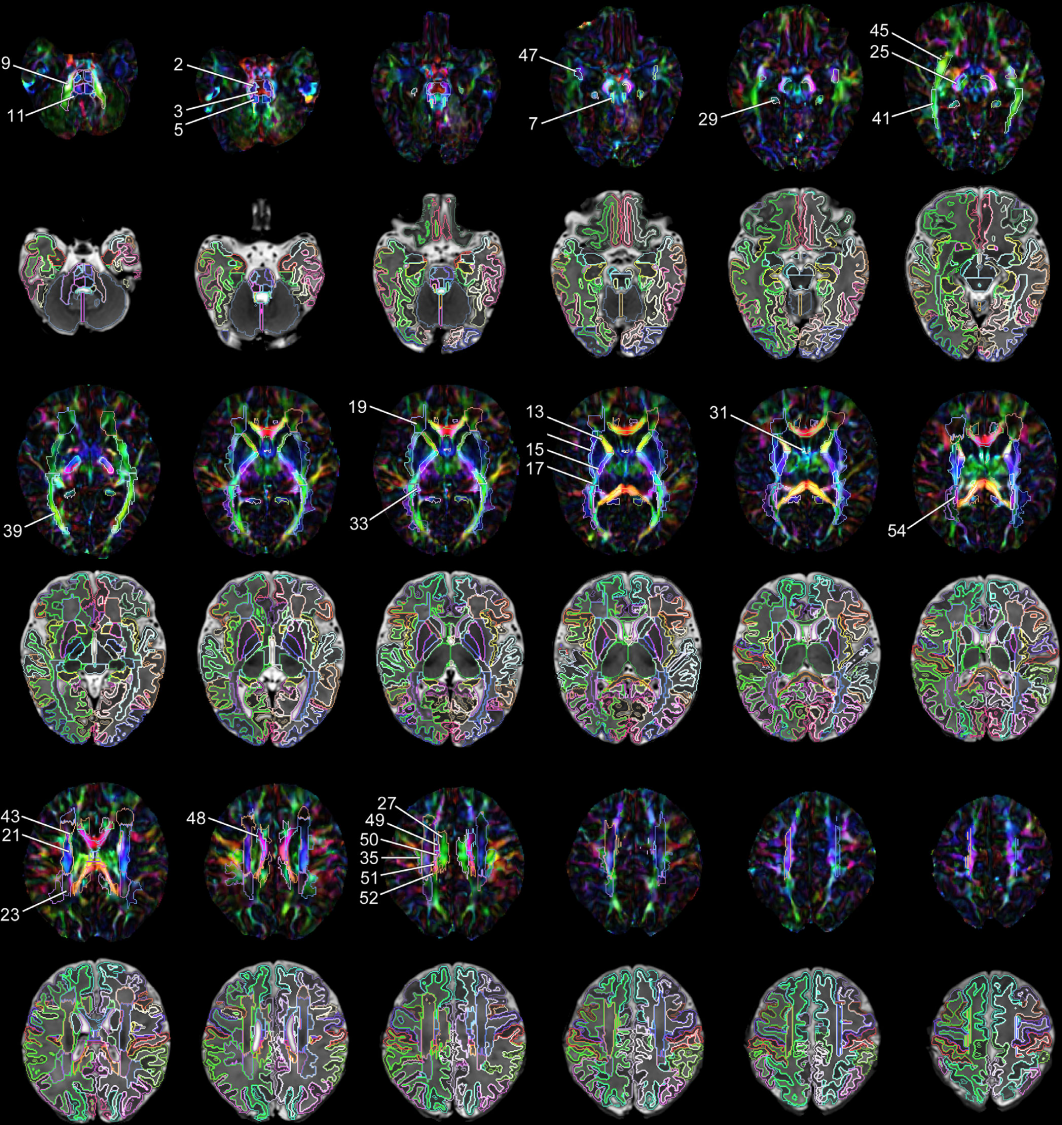
Selected axial slices of M‐CRIB‐WM white matter parcellations (first, third, and fifth rows) overlaid on DEC images; and M‐CRIB‐WM parcellations combined with M‐CRIB 2.0 atlas GM regions (second, fourth, and sixth rows) overlaid on *T*_2_‐weighted images. Images are for a single participant, shown in 5‐slice increments. Images are displayed in radiological orientation. Annotated region numbers correspond to those listed in Table 1. For full detail, see the online, high‐resolution version of this image

**Figure 3:**
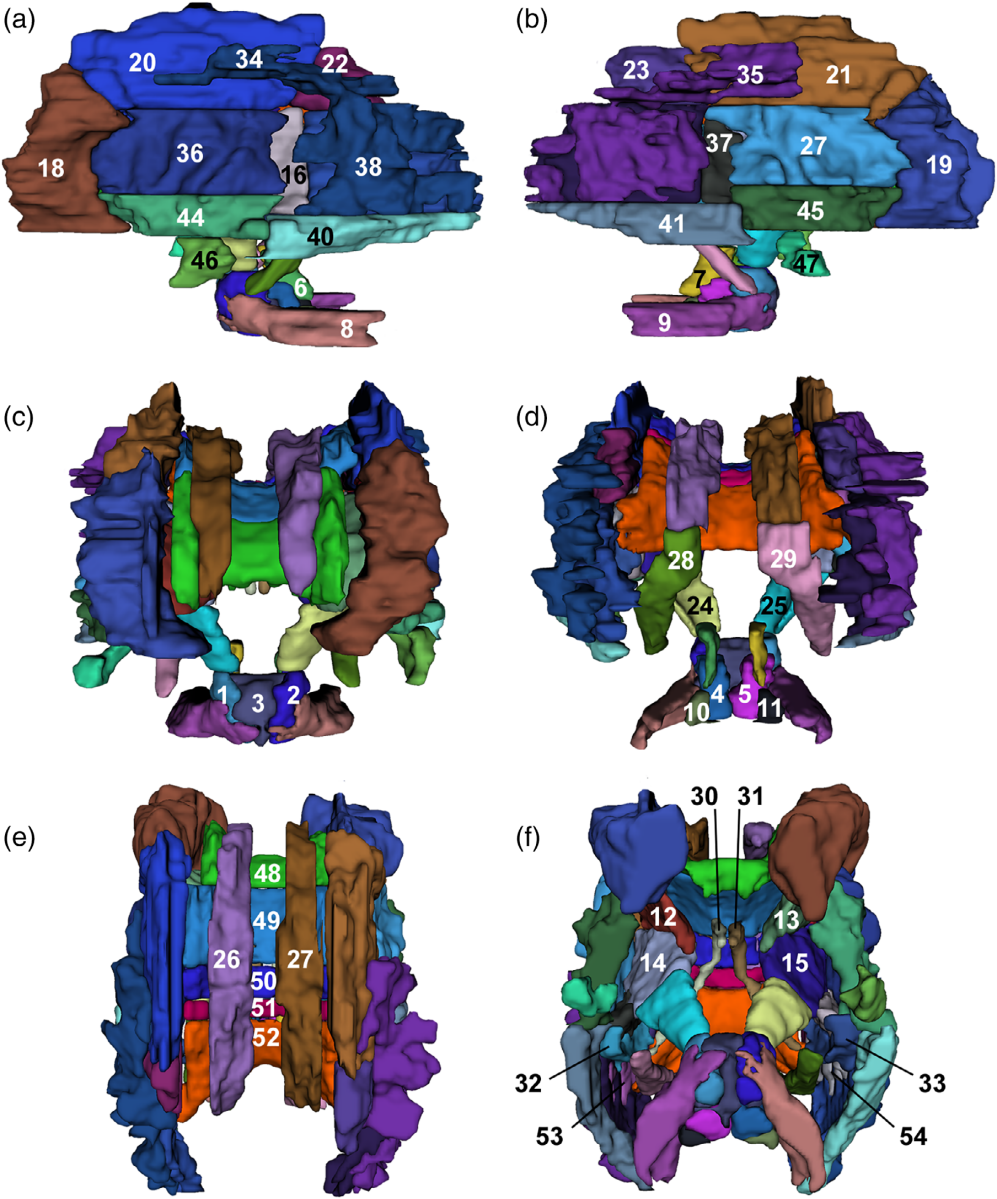
Annotated 3D representation of all white matter parcellations for a single participant. (a) Left hemisphere; (b) right hemisphere; (c) frontal view; (d) occipital view; (e) superior view; (f) inferior view. Surfaces underwent Gaussian smoothing with SD 0.8 mm for display purposes. Labels correspond to structures listed in Table 1. Labels 42 and 43 (not shown) correspond to the superior fronto‐occipital fasciculi, which are not visible from the angles displayed, however, are shown in Figure 2

**Figure 4:**
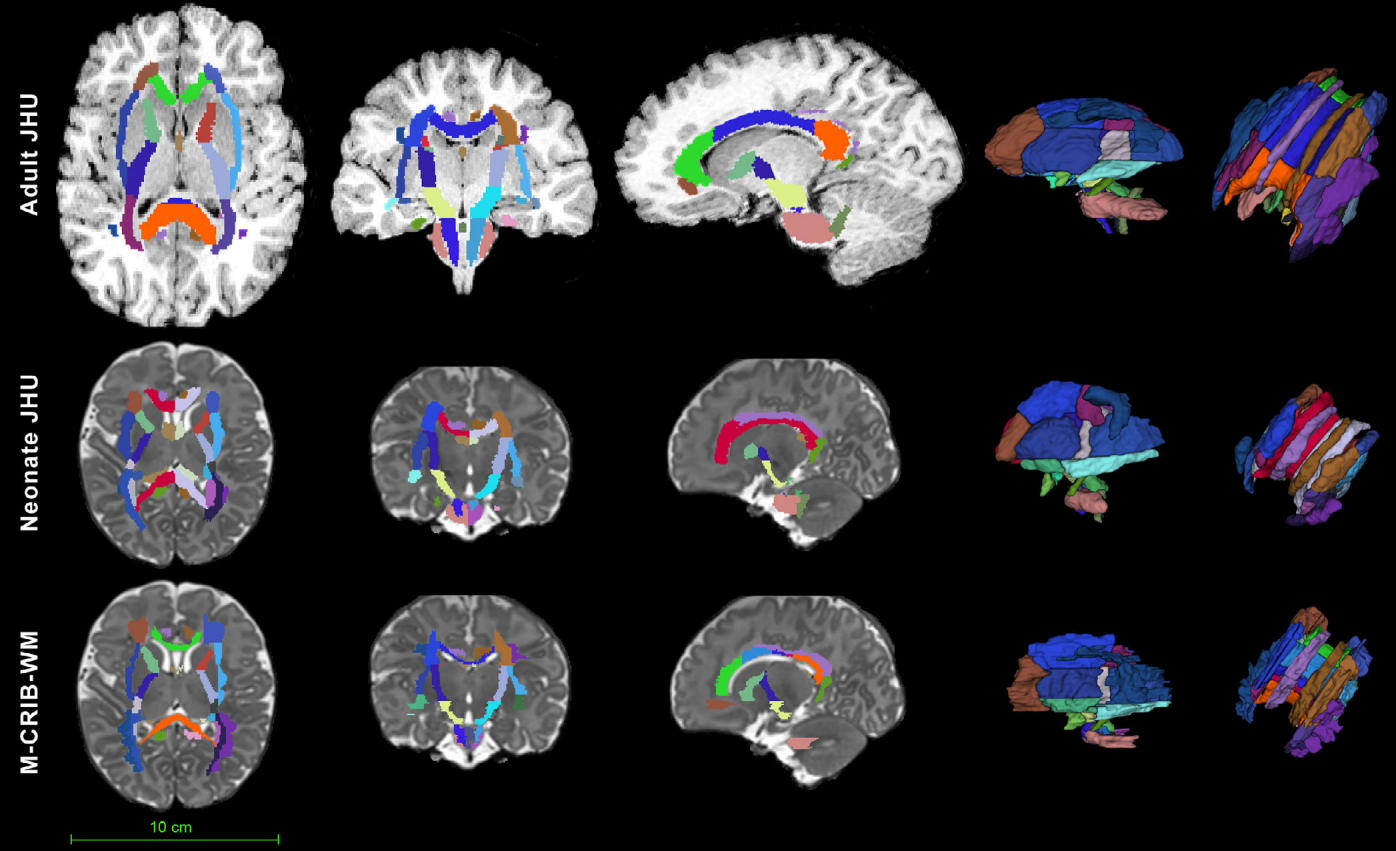
Comparison of the adult JHU white matter atlas, *JHU‐neonate‐SS* atlas, and the M‐CRIB‐WM atlas. Top row: *T*_1_‐weighed image of a healthy 18‐year‐old brain that has been labelled with the adult JHU atlas (Mori & van Zijl, 2007). Middle row: *T*_2_‐weighed image of a single neonatal participant from the M‐CRIB‐WM sample, labelled with the *JHU‐neonate‐SS* (Oishi et al., 2011) atlas. For clarity of comparison, the labels shown are a subset of the 122 *JHU‐neonate‐SS* labels, comprising WM regions and tracts for which corresponding structures have been defined in the M‐CRIB‐WM atlas. Both the neonatal and adult JHU atlas labels were applied by performing nonlinear (symmetric diffeomorphic normalisation) warping of individual structural images to JHU templates using ANTS, and then applying the inverse transformations and warps to the label images. Bottom row: *T*_2_‐weighed image of the same single neonatal participant from the M‐CRIB‐WM sample, with manually parcellated labels overlaid. Relative size of the adult and neonate brains is to scale

The WM extension of the M‐CRIB atlases comprises 24 pairs of left‐ and right‐hemispheric structures, and six single structures, totalling 54 regions. A full list of parcellated WM regions along with their mean volume and standard deviation is included in Table 1. Combining the M‐CRIB‐WM WM regions with the original M‐CRIB (Alexander et al., 2017) whole‐brain atlas, results in a parcellated atlas comprising 154 regions altogether. Combining the WM regions with the M‐CRIB 2.0 (Alexander et al., 2019) whole brain atlas results in 148 parcellated regions. Selected axial slices of the WM parcellations and combined M‐CRIB 2.0 and WM parcellations for a single participant are illustrated in Figure 2. Figure 3 depicts a surface representation of all WM parcellations for a single participant. Figure 4 illustrates volumetric and surface‐based M‐CRIB‐WM parcellations for a single neonatal participant compared with equivalent *JHU‐neonate‐SS* (Oishi et al., 2011) WM labels, and compared with an adult *T*_1_‐weighted brain image labelled using the equivalent adult JHU parcellation scheme (Mori et al., 2008), to illustrate compatibility of the parcellated regions between these time points.

**Table 1:** Complete list of parcellated M‐CRIB‐WM regions and mean volume for each region

Region		Structure	Predominant direction of travel	Mean volume (mm^3^)	*SD* (mm^3^)	Label
Tracts in brainstem	1^a^	CST (L)	SI	238	100	
	2	CST (R)	SI	242	96	
	3	PCT	LR	668	166	
	4	ML (L)	SI	316	110	
	5	ML (R)	SI	296	110	
	6	SCP (L)	SI	99	32	
	7	SCP (R)	SI	97	28	
	8	MCP (L)	AP	682	174	
	9	MCP (R)	AP	696	188	
	10	ICP (L)	AP	74	12	
	11	ICP (R)	AP	75	16	
Projection fibres	12	ALIC (L)	AP	519	56	
	13	ALIC (R)	AP	527	62	
	14	PLIC (L)	SI	759	107	
	15	PLIC (R)	SI	744	104	
	16	RLIC (L)	SI	569	82	
	17	RLIC (R)	SI	619	117	
	18	ACR (L)	AP	2,178	469	
	19	ACR (R)	AP	2,293	511	
	20	SCR (L)	SI	2,956	574	
	21	SCR (R)	SI	2,832	603	
	22	PCR (L)	SI‐AP	1,157	373	
	23	PCR (R)	SI‐AP	1,307	490	
	24	CP (L)	SI	300	56	
	25	CP (R)	SI	309	59	
Association fibres	26	CGC (L)	AP	1,218	213	
	27	CGC (R)	AP	1,259	220	
	28	CGH (L)	SI	514	125	
	29	CGH (R)	SI	508	96	
	30	Fx (L)		197	19	
	31	Fx (R)		178	24	
	32	ST (L)	LR	260	70	
	33	ST (R)	LR	251	55	
	34	SLF (L)	SI	2,290	424	
	35	SLF (R)	SI	2,408	359	
	36	EC (L)	SI	1,319	206	
	37	EC (R)	SI	1,291	206	
	38	PTR (L)	AP	1,563	198	
	39	PTR (R)	AP	1,436	184	
	40	SS (L)	AP	976	147	
	41	SS (R)	AP	983	188	
	42	SFO (L)	AP	148	52	
	43	SFO (R)	AP	142	56	
	44	IFO (L)	AP	689	180	
	45	IFO (R)	AP	706	166	
	46	UFC (L)	SI	159	64	
	47	UFC (R)	SI	176	64	
Commissural fibres	48	CC (I)	LR	1,109	188	
	49	CC (II)	LR	948	137	
	50	CC (III)	LR	346	73	
	51	CC (IV)	LR	211	50	
	52	CC (V)	LR	1,333	358	
	53	TAP (L)	SI	200	71	
	54	TAP (R)	SI	226	79	

*Note:* Labels files containing label indices are provided via the publicly available data set at https://osf.io/mnwv9/

Abbreviations: ACR, anterior corona radiata; AP, anterior–posterior; ALIC, anterior limb of internal capsule; CC, corpus callosum; CGC, cingulum cingular part; CGH, cingulum hippocampal part; CP, cerebral peduncle; EC, external capsule; Fx, Fornix; IFO, inferior fronto‐occipital fasciculus; ICP, inferior cerebellar peduncle; LR, left–right; M‐CRIB‐WM, white matter Melbourne Children's Regional Infant Brain; PCR, posterior corona radiata; PLIC, posterior limb of internal capsule; PTR, posterior thalamic radiation; RLIC, retrolenticular limb of internal capsule; SCR, superior corona radiata; SFO, superior fronto‐occipital fasciculus; SI, superior–inferior; ST, stria terminalis; SS, sagittal stratum; SLF, superior longitudinal fasciculus; UFC, uncinate fasciculus; TAP, tapetum.

a Numbers listed are not label indices.

Figure S1 presents surface representations of all 10 parcellated WM atlases. Figure S2 presents surface representations of combined WM and cortical regions in a single participant. Figure S3 presents selected axial slices of all 10 individual parcellated WM atlases.

The M‐CRIB‐WM individual atlas data sets are publicly available via https://osf.io/mnwv9/, or linked to via https://github.com/DevelopmentalImagingMCRI. The data include parcellated, *T*_2_‐weighed, *T*_1_‐weighed, and DEC NiFTI images for each individual, along with labels files (with corresponding label numbers and descriptions), and basic participant data (sex, age at birth, age at scan) are provided as text files. Individual datasets for a combined atlas comprising both M‐CRIB 2.0 regions (basal ganglia, thalamus, cerebellum, cortex, and other regions) and the current WM regions is also provided. The data are available under a ‘CC‐By Attribution 4.0 International’ creative commons licence, which permits reuse of the data with attribution.

## 4 DISCUSSION

In this work, we present the WM extension to our existing M‐CRIB atlases, the M‐CRIB‐WM. This atlas contains 54 manually parcellated WM regions, in 10 healthy term neonates. The M‐CRIB‐WM has been defined based on high‐quality neonatal DWI and *T*_2_‐weighted data, enabling delineation of the relatively small, detailed structures in the neonatal brain. The use of manual segmentation allowed us to precisely segment structures in each individual.

Manual segmentation remains the best practice for MRI brain parcellation as it allows precise delineation of different brain regions, particularly those with complex or arbitrarily defined boundaries. The WM of the brain, comprising a complex network of neuronal axons, typically has indistinct boundaries between neighbouring WM tracts. For example, the AP‐oriented fibres of the IFO, optic radiation, and PTR traverse through the SS, and cannot be differentiated macroscopically even with meticulous cadaveric fibre dissection techniques (Yasargil, Ture, & Yasargil, 2004). In other instances, the only discernible feature between neighbouring WM tracts is the difference in the dominant fibre orientation. For example, the IFO and UFC are differentiable in the temporal stem because one (IFO) has AP‐oriented fibres, forming the fronto‐occipital connections, whereas the other (UFC) has SI‐oriented fibres and hooks around the temporal stem, forming the fronto‐temporal connections (Choi et al., 2010; Ebeling & von Cramon, 1992; Kier et al., 2004). The WM regions are defined for convenience in anatomical studies. The distinction between neighbouring WM regions is also largely arbitrary. While some WM regions can be defined structurally based on anatomical landmarks (e.g., the CP), there are other WM regions where arbitrary boundaries are unavoidable. In particular, the deep WM regions (ACR, SCR, and PCR) lack clear, recognisable anatomical boundaries between the neighbouring regions. Here, we used imaginary lines drawn perpendicularly from the back edge of the genu of the CC, and the front edge of the splenium of the CC to divide the three portions of the CR, a technique we developed through visual inspection of the JHU adult brain atlas. Although arbitrary, we found this boundary definition reproducible through all subjects.

Terminology that is anatomically clear can reduce ambiguity and bias and allow consistency across different operators. We based our region definitions on those provided for the *JHU‐neonate‐SS atlas* and have further elaborated boundary definitions for the purpose of clarity. For example, the CST label consists of only the ventral pontine portion of this WM tract in the JHU atlases, rather than the entire tract. The resulting detailed parcellation protocols that we have provided for all 54 WM regions, as an elaboration of those provided for the JHU atlases, are a strength of the M‐CRIB‐WM atlas.

A challenge when performing manual parcellation based on DWI is that the dominant colour intensities on the DEC map may be ambiguous, particularly at regions where multiple WM tracts intersect. Our approach to this problem was to develop a discretised Nearest Axis map that indexes only the principal direction (i.e., AP, SI, or LR) in each voxel. This facilitated the identification of a predominant colour on the corresponding DEC map, and thus clarified the principal fibre direction at the boundary between neighbouring regions. This provided a decision solution that enabled us to define anatomical regions with an increased level of certainty. Another strength is that all 10 brains were delineated by a single operator, thus eliminating inter‐rater bias. Although individual bias is a possibility, we were careful to receive detailed feedback on the label boundaries of each brain by both an independent neurosurgery fellow and a paediatric radiologist.

We describe our parcellated WM regions as compatible with the *JHU‐neonate‐SS* regions and adult JHU white matter atlas regions, with the implication that properties of the current regions may be compared with those at later time points labelled with the adult atlas. In doing so, we acknowledge that the boundary definitions have been adapted and further defined here, and that the regions were traced by different operators to those who developed the JHU atlases. As such, there may be some differences in region boundaries or their interpretations by the operators, and parcellation differences based on the resolution, sample participants, and sample size of the data. As described in Section 2, there are also some regions where parcellations differed from those in the *JHU‐neonate‐SS* atlas, and it is necessary to take these into account when designing studies. Users may choose to accept these caveats in view of the advantages this dataset offers.

Considering the time‐consuming nature of the manual segmentation process, the M‐CRIB‐WM atlas is a valuable and unparalleled resource. Whereas other existing detailed parcellated neonatal atlases have been defined in a single subject and/or propagated from other developmental time points, the current atlas comprises 10 individually manually parcellated neonatal brains. A training set of this size has been observed in adult data to be sufficient to represent individual variability in morphology (Croxson et al., 2018; Heckemann et al., 2006), and although there do not appear to be equivalent studies at the neonatal time point, this may at least be a reasonable indication of what may be minimally sufficient in neonates. Parcellation tools can then draw upon labelled multi‐subject training sets by applying multi‐atlas label fusion or emerging deep learning algorithms, which leverage this variability in different ways to optimise labelling accuracy (see reviews by, e.g., Lin & Li, 2019; Makropoulos, Counsell, & Rueckert, 2018). There are multiple available segmentation and parcellation tools for neonatal data that contain pre‐packaged atlases or tissue priors (e.g., Beare et al., 2016; Makropoulos et al., 2018; Makropoulos, Counsell, & Rueckert, 2018; Otsuka et al., 2019; Zollei, Ou, Iglesias, Grant, & Fischl, 2017), although these did not incorporate the M‐CRIB‐WM at the time of publication. To use the current training set to perform parcellation, examples of available tools that allow users to input external volumetric parcellated training sets such as the current atlas include ANTS (Avants et al., 2011), and STAPLE or pSTAPLE (Akhondi‐Asl & Warfield, 2013; Warfield, Zou, & Wells, 2004). These tools rely on diffeomorphic registration and use label fusion algorithms to parcellate data. Future work with the current data may assess the benefits of incorporating DWI in the registration of training and test data, together with *T*_2_‐weighted images. The public release of the current dataset means that it is available for future incorporation as an atlas into existing parcellation tools and may also be used as ground truth for methodological work.

Considering there are equivalent JHU atlases available for younger third trimester time points (Feng et al., 2019) and older time points (Mori et al., 2008; Oishi et al., 2009), our neonatal WM atlas also has the benefit of facilitating longitudinal analyses of neuroimaging metrics from equivalent WM regions. Together with the large range of complementary multi‐parametric neuroimaging tools and techniques available, the combined M‐CRIB and M‐CRIB‐WM atlases will enable detailed structural and microstructural measures to be obtained in an accurate and age‐specific way for major cortical and subcortical regions of the neonatal brain, and now additionally for all major WM tracts and regions. To our knowledge, there has not previously existed a single neonatal atlas encompassing the parcellation of both extensive GM and WM regions to a satisfactory level of detail. Our novel M‐CRIB‐WM atlas, along with the M‐CRIB cortical and subcortical atlases, provides neonatal whole brain MRI coverage of standardised GM and WM parcellations. This addition will greatly benefit the field of infant neuroimaging research.

In summary, we have presented a neonatal WM atlas capturing important WM structural variability unique to, and characteristic of, the neonatal time point. The individual parcellated image maps and structural templates of the M‐CRIB‐WM neonatal atlas, including a complete atlas comprising both M‐CRIB 2.0 regions (basal ganglia, thalamus, cerebellum, cortex, and other regions) and the current WM regions, are publicly available at https://osf.io/mnwv9/ or via https://github.com/DevelopmentalImagingMCRI. This novel atlas will provide extensive neonatal brain coverage with substantial anatomic detail. It will be a valuable resource that will help facilitate investigation of brain structure at the neonatal time point and developmentally across the lifespan.

## CONFLICT OF INTEREST

The authors declare no potential conflict of interest.

## Supporting information


**Figure S1** Surface representation of white matter (WM) parcellations for all 10 M‐CRIB‐WM participants

**Figure S2** Combined surface representation of both M‐CRIB 2.0 cortical parcellation (left hemisphere regions) and M‐CRIB‐WM white matter (WM) parcellations for a single participant. Surfaces underwent Gaussian smoothing with SD 0.8 mm for display purposes

**Figure S3** WM parcellations for all 10 M‐CRIB participants displayed on selected axial slices (approximately 10‐slice increments), overlaid on *T*_*2*_‐weighted images. Images are displayed in radiological orientation. For higher‐resolution detail and corresponding individual DEC images, please see the publicly available dataset at https://osf.io/mnwv9/

## Data Availability

Data availability statement: The data that support the findings of this study are openly available at https://osf.io/mnwv9/ or via https://github.com/DevelopmentalImagingMCRI.

## Appendix A.

Script for co‐registered Nearest Axis map used to aid in boundary delineation.

#! /bin/bash

# diffusion_directions.sh

# creates a 3‐class image with classes defined by closest axis to the principal direction of diffusion in each voxel

# usage:

# ./diffusion_directions.sh

# eg: ./diffusion_directions.sh img_V1.nii.gz img_mask.nii.gz

# output: xyz.nii.gz

# where all voxels w/ intensity 1 = left‐right; intensity 2 = front‐back; intensity 3 = up‐down

dir_image=$1

mask_image=$2

# create x,y,z images

${FSLDIR}/bin/fslmaths $mask_image ‐mul 0 arg0

${FSLDIR}/bin/fslmaths arg0 ‐add 1 arg

${FSLDIR}/bin/fslmerge ‐t xim arg arg0 arg0

${FSLDIR}/bin/fslmerge ‐t yim arg0 arg arg0

${FSLDIR}/bin/fslmerge ‐t zim arg0 arg0 arg

# get distance to each principal direction

echo "measuring…"

${FSLDIR}/bin/fslmaths ${dir_image} ‐abs tmpa

# x‐dir

${FSLDIR}/bin/fslmaths tmpa ‐sub xim ‐sqr tmpx

${FSLDIR}/bin/fslmaths tmpx ‐Tmean ‐mul 3 tmpx

${FSLDIR}/bin/fslmaths tmpx ‐sqrt tmpx

mv tmpx.nii.gz closex.nii.gz

# y‐dir

${FSLDIR}/bin/fslmaths tmpa ‐sub yim ‐sqr tmpy

${FSLDIR}/bin/fslmaths tmpy ‐Tmean ‐mul 3 tmpy

${FSLDIR}/bin/fslmaths tmpy ‐sqrt tmpy

mv tmpy.nii.gz closey.nii.gz

# z‐dir

${FSLDIR}/bin/fslmaths tmpa ‐sub zim ‐sqr tmpz

${FSLDIR}/bin/fslmaths tmpz ‐Tmean ‐mul 3 tmpz

${FSLDIR}/bin/fslmaths tmpz ‐sqrt tmpz

mv tmpz.nii.gz closez.nii.gz

# combine and get index of closest direction

echo "making…"

${FSLDIR}/bin/fslmerge ‐t xyz closex closey closez

${FSLDIR}/bin/fslmaths xyz ‐mul ‐1 ‐Tmaxn ‐add $mask_image xyz

rm ‐rf closex.nii.gz closey.nii.gz closez.nii.gz tmpa.nii.gz arg.nii.gz arg0.nii.gz xim.nii.gz yim.nii.gz zim.nii.gz

echo "done!"
