# Supplementary figures and images for: White matter extension of the Melbourne Children's Regional Infant Brain atlas: M‐CRIB‐WM
Source: Hum Brain Mapp. 2020 Feb 21;41(9):2317–33. doi: 10.1002/hbm.24948 (PMC7267918; doi:10.1002/hbm.24948)

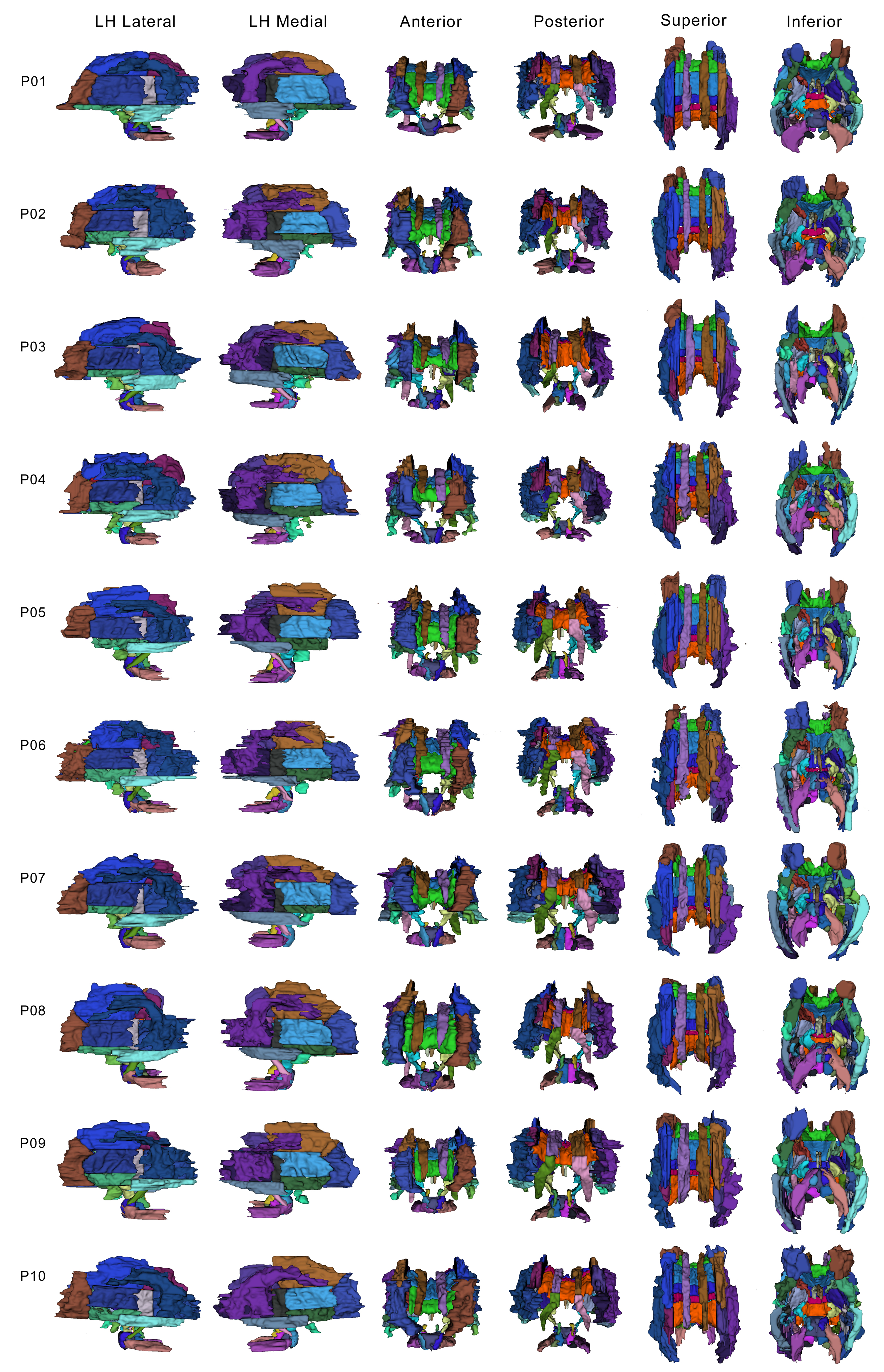

Supplement: Supplementary file 1 — Figure S1 Surface representation of white matter (WM) parcellations for all 10 M‐CRIB‐WM participants [file HBM-41-2317-s001.tif]

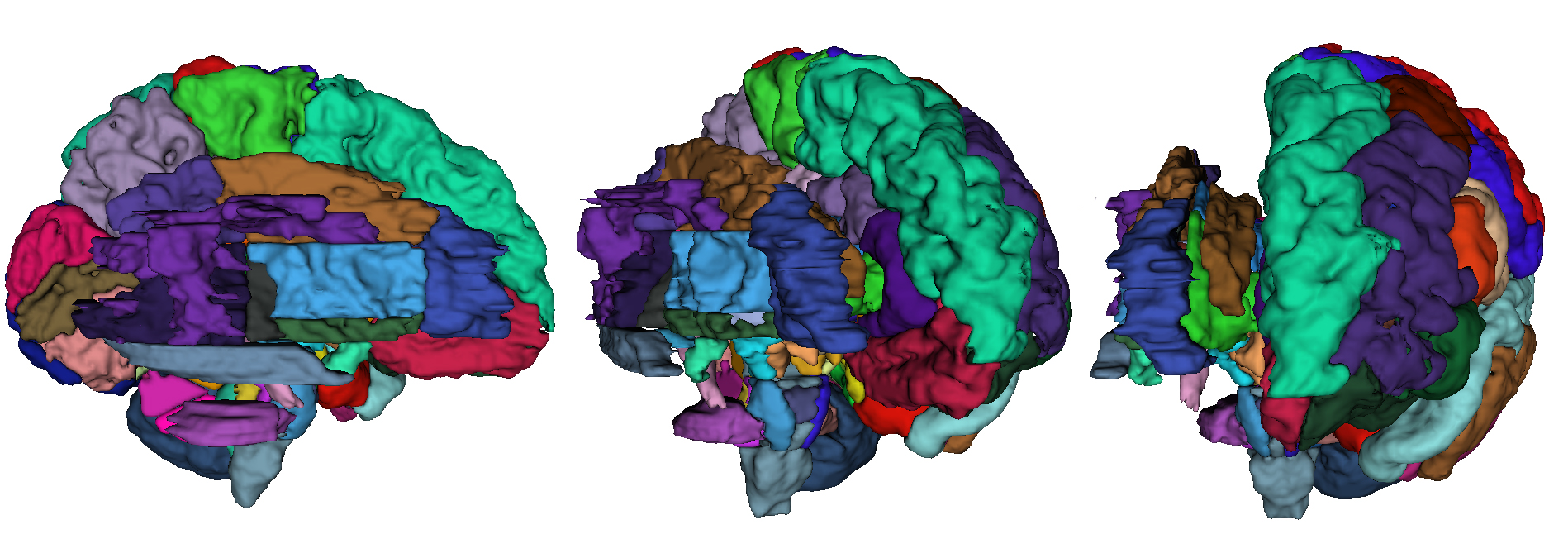

Supplement: Supplementary file 2 — Figure S2 Combined surface representation of both M‐CRIB 2.0 cortical parcellation (left hemisphere regions) and M‐CRIB‐WM white matter (WM) parcellations for a single participant. Surfaces underwent Gaussian smoothing with SD 0.8 mm for display purposes [file HBM-41-2317-s002.tif]

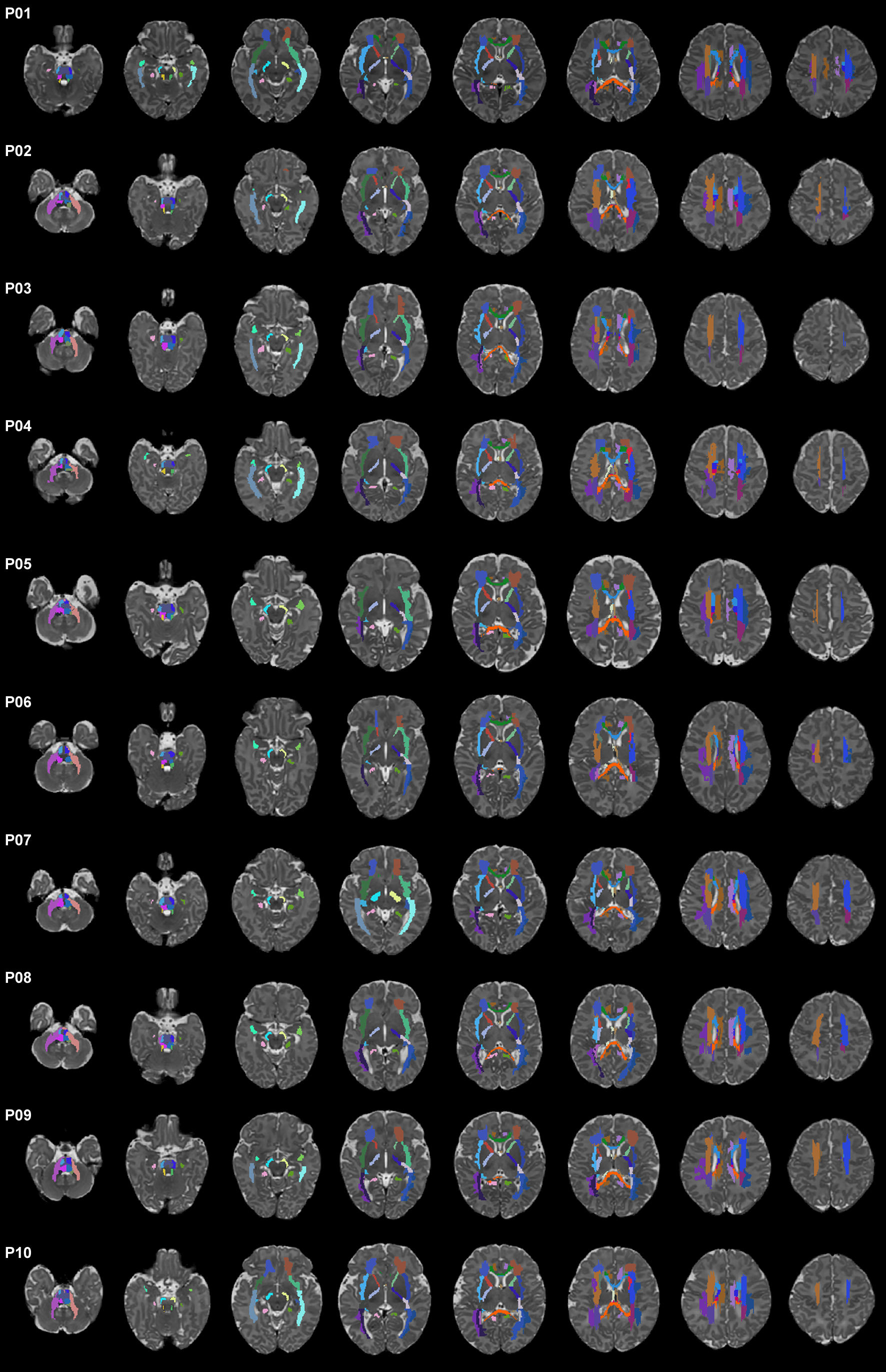

Supplement: Supplementary file 3 — Figure S3 WM parcellations for all 10 M‐CRIB participants displayed on selected axial slices (approximately 10‐slice increments), overlaid on T 2‐weighted images. Images are displayed in radiological orientation. For higher‐resolution detail and corresponding individual DEC images, please see the publicly available dataset at https://osf.io/mnwv9/ [file HBM-41-2317-s003.tif]
